# Camber Angle Inspection for Vehicle Wheel Alignments

**DOI:** 10.3390/s17020285

**Published:** 2017-02-03

**Authors:** Jieh-Shian Young, Hong-Yi Hsu, Chih-Yuan Chuang

**Affiliations:** Institute of Vehicle Engineering, National Changhua University of Education, Changhua County 50007, Taiwan; kotty156@gmail.com (H.-Y.H.); nike81929@yahoo.com.tw (C.-Y.C.)

**Keywords:** accelerometer, camber angle, coordinate transformation, wheel alignment

## Abstract

This paper introduces an alternative approach to the camber angle measurement for vehicle wheel alignment. Instead of current commercial approaches that apply computation vision techniques, this study aims at realizing a micro-control-unit (MCU)-based camber inspection system with a 3-axis accelerometer. We analyze the precision of the inspection system for the axis misalignments of the accelerometer. The results show that the axes of the accelerometer can be aligned to the axes of the camber inspection system imperfectly. The calibrations that can amend these axis misalignments between the camber inspection system and the accelerometer are also originally proposed since misalignments will usually happen in fabrications of the inspection systems. During camber angle measurements, the *x*-axis or *z*-axis of the camber inspection system and the wheel need not be perfectly aligned in the proposed approach. We accomplished two typical authentic camber angle measurements. The results show that the proposed approach is applicable with a precision of $\pm 0.015^{\circ }$ and therefore facilitates the camber measurement process without downgrading the precision by employing an appropriate 3-axis accelerometer. In addition, the measured results of camber angles can be transmitted via the medium such as RS232, Bluetooth, and Wi-Fi.

## 1. Introduction

Vehicle safety and quality are crucial to both manufacturing and maintenance in the automotive industry. Vehicle wheel alignments, including correct inspections and adjustments of wheel characteristic angles, are required [1]. Vehicle wheel alignment becomes essential since the wheel camber angle affects steering controllability and stability, while the wheel toe angle is related to fuel efficiency, tire lifespan, and driving comfort [2]. Misalignments of wheels may effectuate rapid and irregular tire wear. In addition, they may decrease the capability of the vehicle’s handling and safety. The camber angle is defined as the angle between the normal vector of the tire plane and that of the vertical plane viewed from the front of a vehicle. The camber angle has a major influence on the cornering force and on the road handling of the vehicle [3] and therefore plays one of the most significant roles in vehicle handling and safety. In recent decades, technique development for camber angle measurements has received a great amount of attention and has seen tremendous improvement. In brief, measurement techniques moved from mechanical and electromechanical inspection devices to the so-called vision-based systems (2D and 3D). The precision of the measurements by the former technique, which is labor-intensive and time-consuming, has been gross. The latter has improved the precision of the inspections. In general, the precision for camber angle measurements is about $\pm 0.02^{\circ }$ in commercial applications. Furferi et al. have proposed an approach to designing and assessing a machine vision-based system for automatic vehicle wheel alignment [4]. This approach can provide the camber angle without a highly precise application of structured targets on the wheels. Padegaonkar et al. also addressed an idea that implements a contactless inspection of wheel characteristic angles using a 3D stereo-vision technique in order to increase the precision [5]. Baek et al. have utilized point clouds from the range image stream generated by the Microsoft Kinect system, which is equipped with a consumer-grade depth-sensing camera [6].

**Figure 1:**
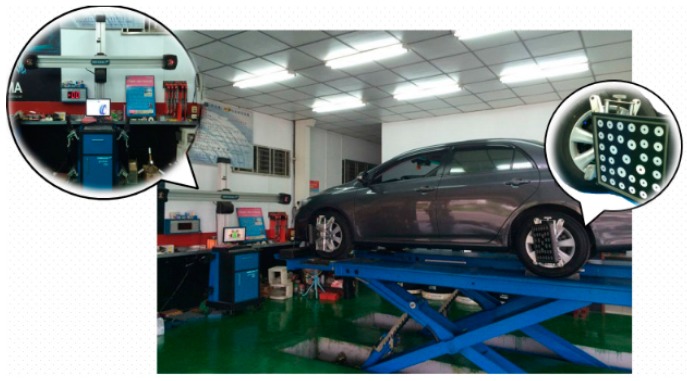
A typical computer vision-based inspection system for wheel alignments.

Figure 1 shows a typical computer vision-based inspection system for wheel alignments. Vision-based systems utilize images captured by video cameras and the image process algorithm to obtain main wheel angles. The target boards have to be mounted on the wheels before applying the system as the vehicle balances on the platform [7,8]. The vehicle must move on the platform for a certain distance while the system operates. Vision-based systems still have some drawbacks although the measurement procedure and the precision have improved. For instance, moving the vehicle on the platform may lead to hazardous conditions. Moreover, measurements take more time when mounting the target boards on the wheels since the mounting procedure, with extremely stable connections, requires a greater amount of attention if more precise results during the movement and rotation of the wheels are desired.

We propose an approach to performing camber angle measurements based on a micro-control unit (MCU). We employed two main components: the MCU and the 3-axis accelerometer. The MCU-based approach makes use of the 3-axis accelerometer to acquire gravity, and applies the coordinate transformation between the camber inspection system and the vehicle. In this approach, perfect alignment for the *x*-axis or the *z*-axis of the camber inspection system and that of the wheel is not necessary since the misalignment angle for the camber inspection system can be compensated by the proposed approach autonomously. Furthermore, the axis misalignment of the accelerometer can also be redressed by an appropriate calibration procedure to increase measurement precision. This paper also analyzes the precision of the inspection system for the axis misalignments of the accelerometer. Two typical authentic camber angle measurements were achieved. The results show that the precision of the MCU-based system is not less than that of the vision-based systems, but the MCU-based system is much cheaper, safer, and more easily operated compared with the vision-based system.

This paper defines the 3-D components in a Cartesian Coordinate *C* as follows:

$${\left(\begin{array}{c} x_{C}\\ y_{C}\\ z_{C} \end{array}\right)}_{C}\;=\;\left[\begin{array}{ccc} {\vec{i}}_{C} & {\vec{j}}_{C} & {\vec{k}}_{C} \end{array}\right]\left[\begin{array}{c} x_{C}\\ y_{C}\\ z_{C} \end{array}\right]$$

where ${\vec{i}}_{C}$, ${\vec{j}}_{C}$, and ${\vec{k}}_{C}$ are the unit vectors of the *x-*, *y-*, and *z*-axes for Coordinate *C*, respectively. That is, ${\left(\begin{array}{ccc} x_{C} & y_{C} & z_{C} \end{array}\right)}_{C}^{T}$ denotes the vector in Coordinate *C* and ${\left[\begin{array}{ccc} x_{C} & y_{C} & z_{C} \end{array}\right]}^{T}$ denotes the 3-by-1 matrix according to the vector ${\left(\begin{array}{ccc} x_{C} & y_{C} & z_{C} \end{array}\right)}_{C}^{T}$. *C* can be vehicles, wheels, the accelerometer, or the camber inspection system in this paper.

## 2. The Coordinate Transformation between Vehicle and Camber Inspection System

**Figure 2:**
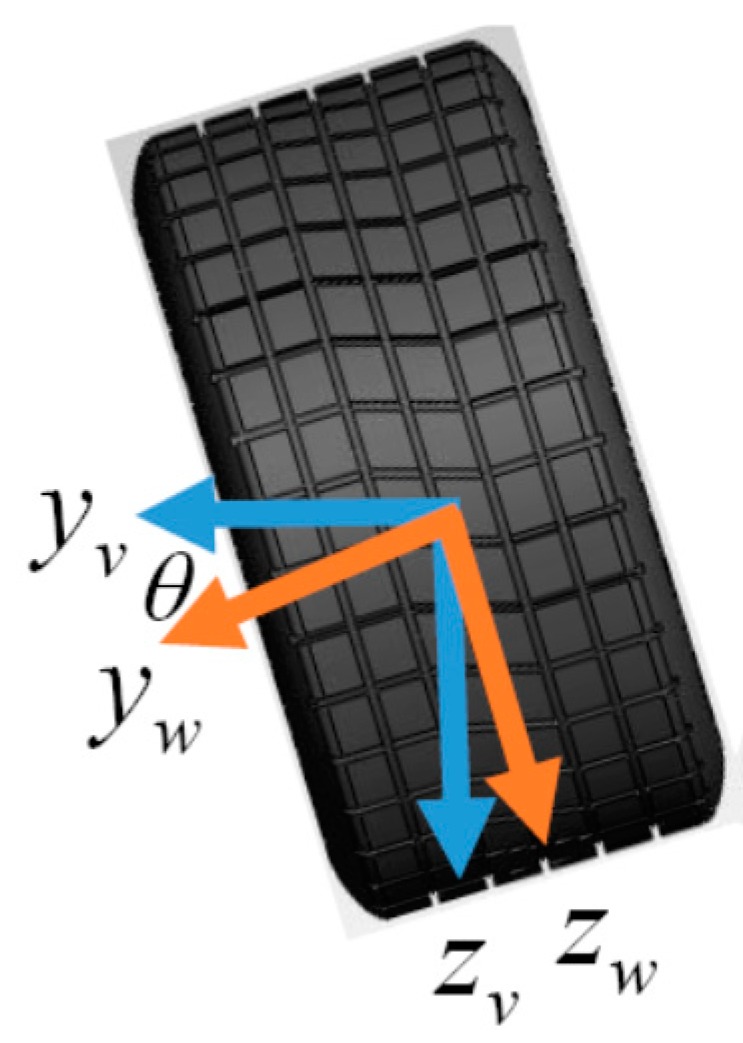
Front view of the vehicle and wheel coordinates with the pitch (camber) angle.

**Figure 3:**
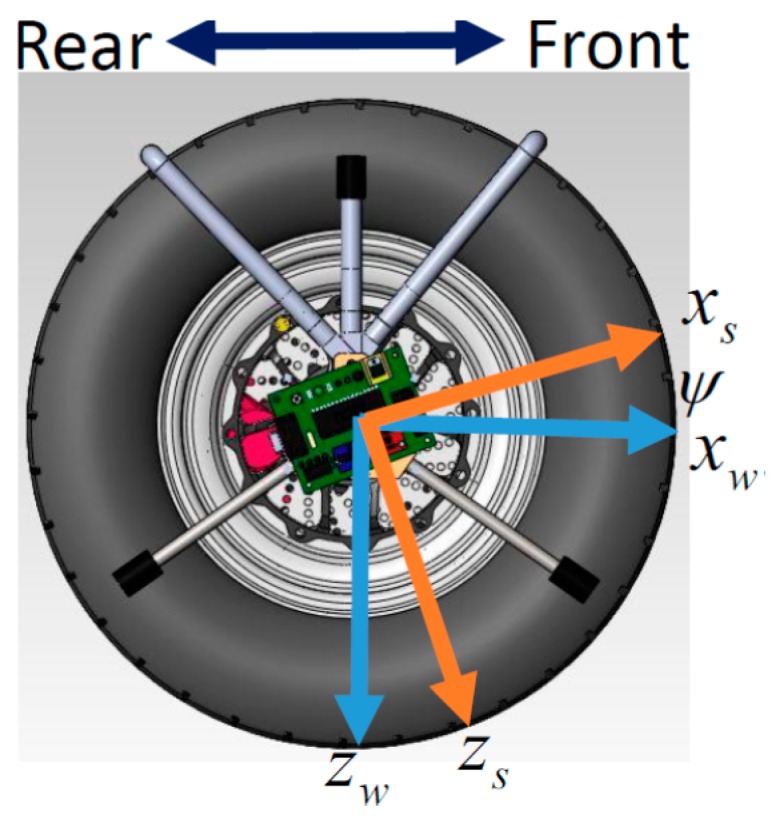
Right side view of the front-right wheel and the camber inspection system with yaw angles.

The camber inspection system can sense the local gravity (not always equal to 1 g = 9.8 m/s^2^) in the camber inspection system coordinate (Coordinate *S*), while the local gravity is only in downward of the vehicle coordinate (Coordinate *V*). The coordinate transformation between Coordinates *V* and *S* will facilitate computations for the camber measurement since the local gravity is the same vector with different representations according to Coordinates *V* and *S*. Figure 2 shows Coordinate *V,* where *x-*, *y-*, and *z*-axes of Coordinate *V* are defined as forward, rightward, and downward, respectively. The *x-*, *y-*, and *z*-axes of Coordinate *S* aligned with the main axes of the system are shown in Figure 3. The Euler angles between Coordinates *V* and *S* including pitch and yaw angles are necessary in coordinate transformations. The camber angle is definitely the pitch angle, θ. In addition, the yaw angle, ψ, is the misaligned angle between the *x*-axis of Coordinate *S* and the wheel coordinate (Coordinate *W*). The pitch angle and the yaw angle are rotated by the *x*-axis of Coordinate *V* and by the *y*-axis of Coordinate *W*, respectively. Intuitively, the origins of Coordinates *V*, *W*, and *S* are the same. An object, e.g., the gravity, can be in different coordinates. That is,

$${\left(\begin{array}{c} x_{V}\\ y_{V}\\ z_{V} \end{array}\right)}_{V}={\left(\begin{array}{c} x_{W}\\ y_{W}\\ z_{W} \end{array}\right)}_{W}$$

or

$$\left[\begin{array}{ccc} {\vec{i}}_{V} & {\vec{j}}_{V} & {\vec{k}}_{V} \end{array}\right]\left[\begin{array}{c} x_{V}\\ y_{V}\\ z_{V} \end{array}\right]=\left[\begin{array}{ccc} {\vec{i}}_{W} & {\vec{j}}_{W} & {\vec{k}}_{W} \end{array}\right]\left[\begin{array}{c} x_{W}\\ y_{W}\\ z_{W} \end{array}\right].$$

According to the definition of the coordinates in Figure 2, the transformation between Coordinates *V* and *W* is as follows:

(1) $$\left[\begin{array}{c} x_{V}\\ y_{V}\\ z_{V} \end{array}\right]=\left[\begin{array}{ccc} 1 & 0 & 0\\ 0 & \cos\theta & -\sin\theta \\ 0 & \sin\theta & \cos\theta \end{array}\right]\left[\begin{array}{c} x_{W}\\ y_{W}\\ z_{W} \end{array}\right].$$

Similarly, from the definition in Figure 3, the coordinate transformation between the Coordinates *W* and *S* can be

(2) $$\left[\begin{array}{c} x_{W}\\ y_{W}\\ z_{W} \end{array}\right]=\left[\begin{array}{ccc} \cos\psi & 0 & \sin\psi \\ 0 & 1 & 0\\ -\sin\psi & 0 & \cos\psi \end{array}\right]\left[\begin{array}{c} x_{S}\\ y_{S}\\ z_{S} \end{array}\right]$$

where θ and ψ are the angle rotated from Coordinate *V* to Coordinate *W* and the angle rotated from Coordinate *W* to Coordinate *S*, respectively. Substituting Equation (2) into Equation (1), the coordinate transformation between Coordinates *V* and *S* become

(3) $$\left[\begin{array}{c} x_{V}\\ y_{V}\\ z_{V} \end{array}\right]=\left[\begin{array}{ccc} \cos\psi & 0 & \sin\psi \\ \sin\theta \sin\psi & \cos\theta & -\sin\theta \cos\psi \\ -\cos\theta \sin\psi & \sin\theta & \cos\theta \cos\psi \end{array}\right]\left[\begin{array}{c} x_{S}\\ y_{S}\\ z_{S} \end{array}\right].$$

## 3. Camber Angle Inspection and Error Analysis

### 3.1. Camber Angle Inspection

The local gravity is only the acceleration sensed by the accelerometer. It is along the *z*-axis of Coordinate *V* when the vehicle is steady on the horizontal platform during camber angle measurements, i.e., $\vec{a}=g{\vec{k}}_{V}.$ The accelerometer can acquire the acceleration $\vec{a}=a_{x}{\vec{i}}_{S}+a_{y}{\vec{j}}_{S}+a_{z}{\vec{k}}_{S}$, where $a_{x}$, $a_{y}$, and $a_{z}$ are the accelerations of the *x*, *y*, and *z* components in Coordinate *S*, respectively. In this case,

$${\left(\begin{array}{c} 0\\ 0\\ g \end{array}\right)}_{V}={\left(\begin{array}{c} a_{x}\\ a_{y}\\ a_{z} \end{array}\right)}_{S}$$

Equation (3) yields

(4) $$\psi =-\tan^{-1}\frac{a_{x}}{a_{z}}$$

and

(5) $$\theta =\tan^{-1}\frac{a_{y}}{a_{z}\cos\psi -a_{x}\sin\psi }.$$

Substituting Equation (4) into Equation (5), the camber angle becomes

(6) $$\theta =\tan^{-1}\frac{a_{y}}{\sqrt{a_{x}^{2}+a_{z}^{2}}}.$$

From the proposed approach, the camber angle measurement is independent of the misaligned angle, ψ, between Coordinate *S* and Coordinate *W*. Moreover, the misaligned angle can be evaluated from Equation (4). The camber angle, θ, of a wheel can be directly evaluated from Equation (6), as the accelerations sensed from the accelerometer are ready, whether the *x*-axis of Coordinate *S* and that of Coordinate *W* are perfectly aligned in the same direction or not. That is, the proposed camber angle measurement approach will allow technicians to easily operate camber angle measurements by the MCU-based system, without additional technical training. However, in application, this study suggests that the *x*-axis of Coordinate *S* may be close to that of Coordinate *W* due to the computational accuracy from the MCU, even though perfect alignment is not necessary in Equation (6).

### 3.2. Calibrations for the Misaligned Axes between the Camber Inspection System and the Accelerometer

The axis misalignments between the camber inspection system and the accelerometer will usually happen in fabrications of MCU-based camber inspection systems. The misalignments will deteriorate the precisions of the camber angle inspections. The calibrations for these misalignments become indispensable. The calibrations are the coordinate transformation between them distinctly. Let $x_{k,i}$, $y_{k,i}$, and $z_{k,i}$ denote the measured values from the accelerometer, where *k,* which can be *x*, *y*, or *z,* is the index for the *k*-axis calibration of the system; *i*, ranging from 1 to *N,* is the index of the *i*-th set data; *N* is the total number of measured data. ${\bar{x}}_{k}$, ${\bar{y}}_{k}$, and ${\bar{z}}_{k}$ denote the optimal estimated values respectively for $x_{k,i}$, $y_{k,i}$, and $z_{k,i}$ of *N* data with the least square performance constrained by the average gravity. Thus, the performance index is

(7) $$J=\sum_{i=1}^{N}{\left({\bar{x}}_{k}-x_{k,i}\right)}^{2}+\sum_{i=1}^{N}{\left({\bar{y}}_{k}-y_{k,i}\right)}^{2}+\sum_{i=1}^{N}{\left({\bar{z}}_{k}-z_{k,i}\right)}^{2}$$

with the average gravity constraint

(8) $$g({\bar{x}}_{k},{\bar{y}}_{k},{\bar{z}}_{k})={\bar{x}}_{k}^{2}+{\bar{y}}_{k}^{2}+{\bar{z}}_{k}^{2}-\frac{{\left(\sum_{i=1}^{N}\sqrt{x_{k,i}^{2}+y_{k,i}^{2}+z_{k,i}^{2}}\right)}^{2}}{N^{2}}=0.$$

From Equations (7) and (8), it is a constrained optimization problem, and the Lagrange function can be as follows:

(9) $$\begin{array}{l} L({\bar{x}}_{k},{\bar{y}}_{k},{\bar{z}}_{k},\lambda_{k})=\sum_{i=1}^{N}{\left({\bar{x}}_{k}-x_{k,i}\right)}^{2}+\sum_{i=1}^{N}{\left({\bar{y}}_{k}-y_{k,i}\right)}^{2}+\sum_{i=1}^{N}{\left({\bar{z}}_{k}-z_{k,i}\right)}^{2}\\ +\lambda_{k}({\bar{x}}_{k}^{2}+{\bar{y}}_{k}^{2}+{\bar{z}}_{k}^{2}-\frac{{\left(\sum_{i=1}^{N}\sqrt{x_{k,i}^{2}+y_{k,i}^{2}+z_{k,i}^{2}}\right)}^{2}}{N^{2}}) \end{array}$$

where $\lambda_{k}$ is the Lagrange multiplier. The optimality conditions of Equation (9) are $\frac{\partial L}{\partial {\bar{x}}_{k}}=0$, $\frac{\partial L}{\partial {\bar{y}}_{k}}=0$, $\frac{\partial L}{\partial {\bar{z}}_{k}}=0$, and $\frac{\partial L}{\partial \lambda_{k}}=0$; i.e.,

(10) $${\bar{x}}_{k}=\frac{\sum_{i=1}^{N}x_{k,i}}{N+\lambda_{k}}$$

(11) $${\bar{y}}_{k}=\frac{\sum_{i=1}^{N}y_{k,i}}{N+\lambda_{k}}$$

and

(12) $${\bar{z}}_{k}=\frac{\sum_{i=1}^{N}z_{k,i}}{N+\lambda_{k}}.$$

Substituting Equations (10)–(12) into Equation (8), we obtain

(13) $$\lambda_{k}=N\left(\frac{\sqrt{{\left(\sum_{i=1}^{N}x_{k,i}\right)}^{2}+{\left(\sum_{i=1}^{N}y_{k,i}\right)}^{2}+{\left(\sum_{i=1}^{N}z_{k,i}\right)}^{2}}}{\sum_{i=1}^{N}\sqrt{x_{k,i}^{2}+y_{k,i}^{2}+z_{k,i}^{2}}}-1\right).$$

According to Equation (13),

(14) $${\bar{x}}_{k}=\frac{\sum_{i=1}^{N}x_{k,i}}{N}\frac{\sum_{i=1}^{N}\sqrt{x_{k,i}^{2}+y_{k,i}^{2}+z_{k,i}^{2}}}{\sqrt{{\left(\sum_{i=1}^{N}x_{k,i}\right)}^{2}+{\left(\sum_{i=1}^{N}y_{k,i}\right)}^{2}+{\left(\sum_{i=1}^{N}z_{k,i}\right)}^{2}}}$$

(15) $${\bar{y}}_{k}=\frac{\sum_{i=1}^{N}y_{k,i}}{N}\frac{\sum_{i=1}^{N}\sqrt{x_{k,i}^{2}+y_{k,i}^{2}+z_{k,i}^{2}}}{\sqrt{{\left(\sum_{i=1}^{N}x_{k,i}\right)}^{2}+{\left(\sum_{i=1}^{N}y_{k,i}\right)}^{2}+{\left(\sum_{i=1}^{N}z_{k,i}\right)}^{2}}}$$

and

(16) $${\bar{z}}_{k}=\frac{\sum_{i=1}^{N}z_{k,i}}{N}\frac{\sum_{i=1}^{N}\sqrt{x_{k,i}^{2}+y_{k,i}^{2}+z_{k,i}^{2}}}{\sqrt{{\left(\sum_{i=1}^{N}x_{k,i}\right)}^{2}+{\left(\sum_{i=1}^{N}y_{k,i}\right)}^{2}+{\left(\sum_{i=1}^{N}z_{k,i}\right)}^{2}}}.$$

**Figure 4:**
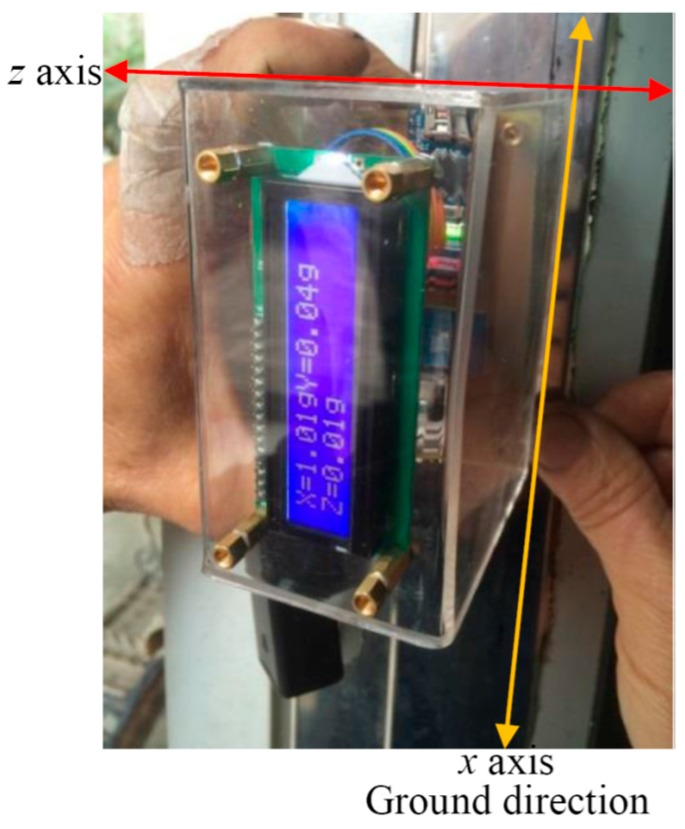
The *x*-axis calibration of the camber inspection system, whose back plane leaned on a vertical plane with the *x*-axis pointing to the ground.

For instance, during *x*-axis calibration of the camber inspection system, the *x*-axis directs to the ground vertically while the system leans on a vertical plane, as shown in Figure 4. The measured data of the 3-axis acceleration from the accelerometer can be collected sequentially. From Equations (14)–(16), it yields

(17) $${\left(\begin{array}{c} 1\\ 0\\ 0 \end{array}\right)}_{S}={\left(\begin{array}{c} x_{x}\\ y_{x}\\ z_{x} \end{array}\right)}_{A}$$

where *A* denotes the accelerometer (Coordinate *A*), $x_{x}=\frac{{\bar{x}}_{x}}{\sqrt{{\bar{x}}_{x}^{2}+{\bar{y}}_{x}^{2}+{\bar{z}}_{x}^{2}}}$, $y_{x}=\frac{{\bar{y}}_{x}}{\sqrt{{\bar{x}}_{x}^{2}+{\bar{y}}_{x}^{2}+{\bar{z}}_{x}^{2}}}$, and $z_{x}=\frac{{\bar{z}}_{x}}{\sqrt{{\bar{x}}_{x}^{2}+{\bar{y}}_{x}^{2}+{\bar{z}}_{x}^{2}}}$. The *z*-axis calibration of the system can follow the same procedure; i.e.,

(18) $${\left(\begin{array}{c} 0\\ 0\\ 1 \end{array}\right)}_{S}={\left(\begin{array}{c} x_{z}\\ y_{z}\\ z_{z} \end{array}\right)}_{A}.$$

Since the *x*-axis and *z*-axis are on the vertical plane, they can be calibrated for the sake of the back plane of the camber inspection system. $x_{x}x_{z}+y_{x}y_{z}+z_{x}z_{z}=0$ (the inner product operation results from Equations (17) and (18)) if the calibrations of the *x*-axis and *z*-axis are fulfilled. Thus,

$${\left(\begin{array}{c} 0\\ 1\\ 0 \end{array}\right)}_{S}={\left(\begin{array}{c} x_{y}\\ y_{y}\\ z_{y} \end{array}\right)}_{A}$$

where ${\left(\begin{array}{ccc} x_{y} & y_{y} & z_{y} \end{array}\right)}_{A}^{T}={\left(\begin{array}{ccc} x_{z} & y_{z} & z_{z} \end{array}\right)}_{A}^{T}\times {\left(\begin{array}{ccc} x_{x} & y_{x} & z_{x} \end{array}\right)}_{A}^{T}$. Consequently, if

$${\left(\begin{array}{c} a_{x}\\ a_{y}\\ a_{z} \end{array}\right)}_{S}={\left(\begin{array}{c} a_{x}^{a}\\ a_{y}^{a}\\ a_{z}^{a} \end{array}\right)}_{A}$$

then

(19) $$\left[\begin{array}{c} a_{x}\\ a_{y}\\ a_{z} \end{array}\right]={\left[\begin{array}{ccc} x_{x} & x_{y} & x_{z}\\ y_{x} & y_{y} & y_{z}\\ z_{x} & z_{y} & z_{z} \end{array}\right]}^{-1}\left[\begin{array}{c} a_{x}^{a}\\ a_{y}^{a}\\ a_{z}^{a} \end{array}\right].$$

That is, $a_{x}^{a}$, $a_{y}^{a}$, and $a_{z}^{a}$, which can be transformed respectively to the corresponding accelerations of the camber inspection system, are the measured data from the accelerometer. Accordingly, the precise calibrations can eliminate the effects of the misalignment for the axes of the system and the accelerometer. Furthermore, the camber angle can be measured from Equation (6) with the raw measured data by the accelerometer coordinate transformed in Equation (19).

### 3.3. Measuring Error Analysis

The measuring error is one of the most significant issues in camber angle measurements. The error analysis can facilitate dissecting the effects on all possible attitudes of the equipped accelerometer in the camber inspection system. Furthermore, the results can provide suggestions for better attitudes to improve the precision of the camber inspection system. It becomes crucial for the proposed approach. From Equation (6), the differentiation of the camber angle, dθ, can be as follows:

(20) $$d\theta =\frac{1}{\sqrt{a_{x}^{2}+a_{y}^{2}+a_{z}^{2}}}(-\sin\theta \sin\psi da_{x}+\cos\theta da_{y}-\sin\theta \cos\psi da_{z})$$

where $da_{x}$, $da_{y}$, and $da_{z}$ are the resolutions of the accelerometer for *x-*, *y-*, and *z*-axes, respectively. The magnitude of the acceleration sensed from the camber inspection system is equal to the local gravity approximate to 1 g in most cases; i.e.,

$$a_{x}^{2}+a_{y}^{2}+a_{z}^{2}≅g^{2}.$$

Thus, Equation (20) turns to

(21) $$d\theta ≅\frac{1}{g}(-\sin\theta \sin\psi da_{x}+\cos\theta da_{y}-\sin\theta \cos\psi da_{z}).$$

In the ill conditions of the accelerometer measurements in which all the signs of the right terms in Equation (21) are the same, Equation (21) becomes

(22) $$\left|d\theta \right|≅\frac{1}{g}(\left|\sin\theta \sin\psi da_{x}\right|+\left|\cos\theta da_{y}\right|+\left|\sin\theta \cos\psi da_{z}\right|)$$

since all the terms on the right side of Equation (21) are in the same sign possibly. Equation (22) elucidates that the camber measuring error highly depends on the resolutions of all accelerometer axes. From Equation (22), the minimal measuring error in the ill condition is equal to the ratio of $da_{y}$ and the gravity as $\theta =0^{\circ }$; i.e.,

(23) $$\underset{\theta ,\psi }{\min}\left|d\theta \right|=\frac{\left|da_{y}\right|}{g}.$$

**Figure 5:**
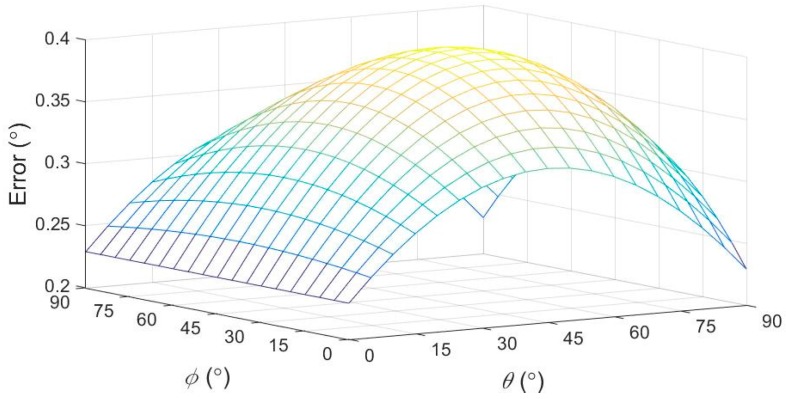
From Equation (22), the camber measuring errors due to the accelerometer resolutions for different θ and ψ values in the ill conditions of the accelerometer measurements.

In general, the 3-axis resolutions of an accelerometer are not the same. Equation (23) can be construed as implying that, to reduce the measuring error, the best resolution axis of an accelerometer should be aligned close to the *y*-axis of Coordinate *S*. In addition, the measuring error in Equation (22) also depends on θ (the camber angle) and ψ (the misaligned angle), or the attitude of Coordinate *S* related to Coordinate *V*. Based on Equation (22), Figure 5 sketches the camber angle measuring errors for different θ and ψ values in the case of $da_{x}=da_{y}=da_{z}=\pm \;4mg$ (*g* is the acceleration of gravity) in the ill conditions of the accelerometer measurements, or the same signs of the terms on the right side of Equation (21).

From Equation (23), the minimal measuring error approximates to $0.2292^{\circ }$ (0.0040 rad) as $\theta =0^{\circ }$. The camber angle is around 0, i.e., $\theta ≅0^{\circ }$, in authentic measurements. For instance, the error will become $0.2393^{\circ }$ as $\theta =2^{\circ }$ and $\psi =20^{\circ }$ in the ill conditions, although the effect is not veritably significant in camber angle measurements. In addition, based on Equation (22), we also suggest that the *x*-axis of Coordinate *S* should be close to that of Coordinate *W* to obtain an improved measurement of the camber angle since the error depends on the misaligned angle, ψ, as $\theta \neq 0^{\circ }$. These types of errors will happen not only in the measurement process but also in the installation of the accelerometer, with misalignments between the axes of the camber inspection system and those of the accelerometer. Technically, an operator can easily install an accelerometer in the MCU-based camber inspection system with $\psi \leq 20^{\circ }$.

## 4. Authentic Camber Angle Inspections with Precision Improvement

**Figure 6:**
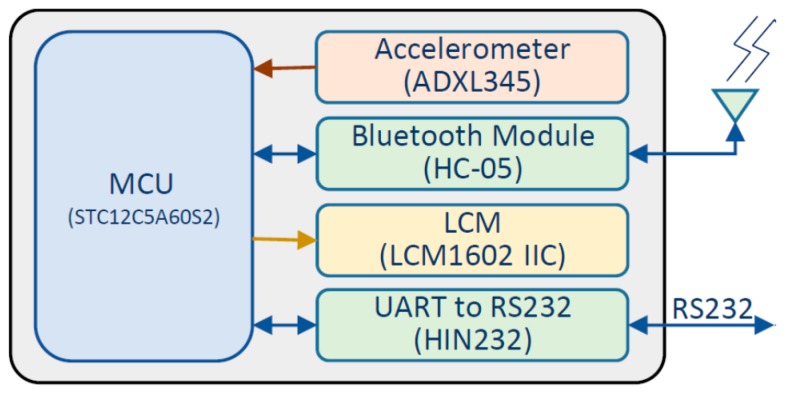
The function block diagram of the MCU-based camber inspection system.

**Figure 7:**
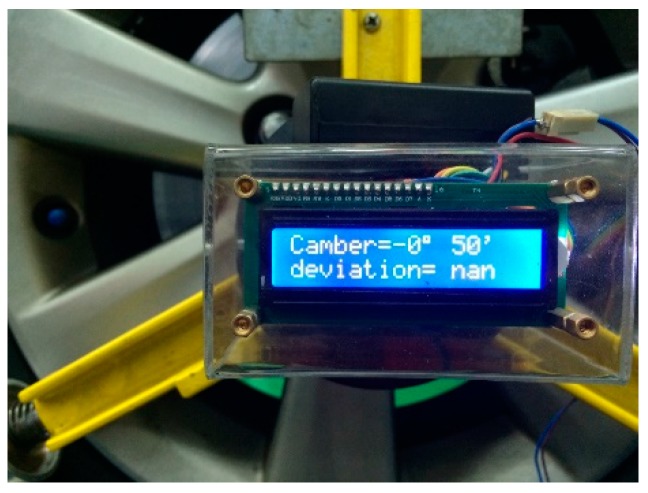
The MCU-based camber inspection system.

We mainly employed the MCU and the 3-axis accelerometer to realize the proposed approach. The accelerometer acquires the gravity in three axes while the MCU calculates the camber angle by the proposed approach. Figure 6 shows the function block diagram of the MCU-based camber inspection system. The type numbers of the MCU, the accelerometer, the bluetooth module, the liquid crystal display module (LCM), and the UART chip are STC12C5A60S2, ADXL345, HC-05, LCM1602 IIC, and HIN232, respectively. This paper implements the MCU-based camber inspection system, as shown in Figure 7, for the pilot study and the verifications of the proposed approach.

The measuring error analysis suggests that the axes of the accelerometer aligned to the axes of the camber inspection system can meliorate inspection precision. The theoretic minimal measuring error of the camber inspection system is more than $0.2292^{\circ }$ in ill conditions for the specified resolutions, or $da_{x}=da_{y}=da_{z}=\pm \;4mg$, of the accelerometer without signal filtering. The precision of the commercial vision-based system is about $0.02^{\circ }$. The precision of the realized camber inspection system is not available for camber angle measurements without filtering, although in most cases the measuring errors are within $0.2^{\circ }$. The calibrations for at least the *x-* and *z*-axes is essential for increasing the precision of the camber inspection system due to the misalignments between the axes of the system and those of the accelerometer as fabrications of the systems. According to the many authentic calibration experiments, we empirically suggest that, for better precision, the total number of measured data is more than 50 as calibrating for each axis.

**Figure 8:**
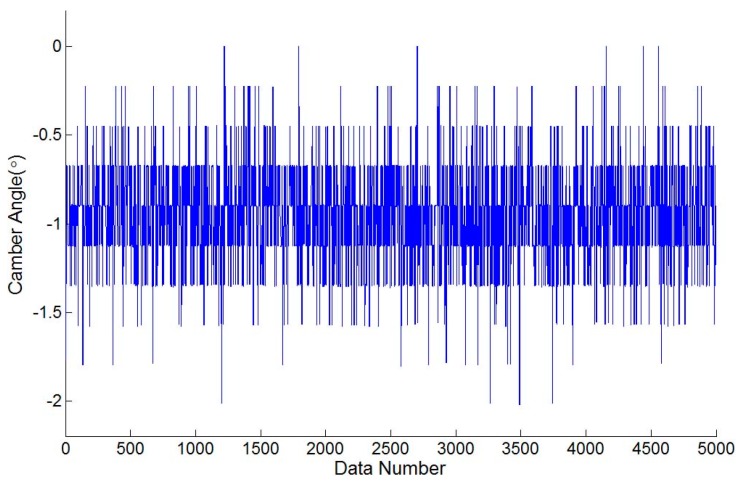
The raw measured data evaluated from the MCU-based system with a camber angle of $-00^{\circ }54\prime$.

**Figure 9:**
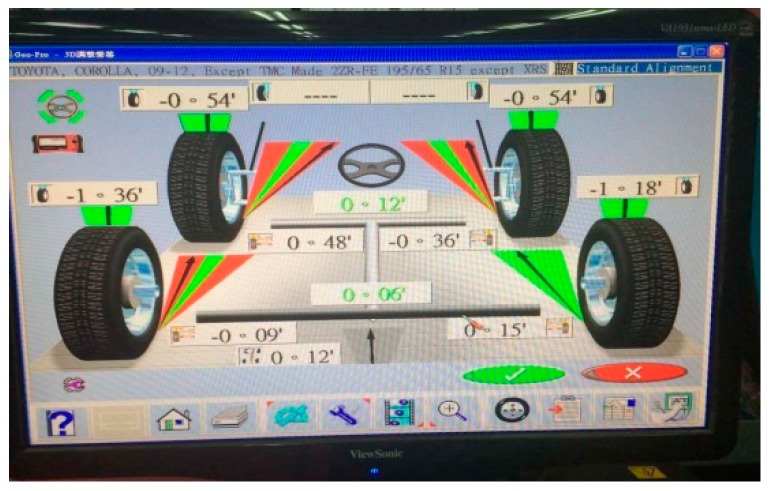
The camber measuring results from a traditional vision-based system.

During an authentic camber angle inspection, for instance, Figure 8 shows the raw measured data of a front-right wheel from a vehicle with a camber angle of $-0.9000^{\circ }\;(-0^{\circ }54\prime )$ evaluated by the vision-based system as shown in Figure 9. The evaluated camber angles are available if the standard deviations of constant number data are within a specified value, e.g., $0.005^{\circ }$. The deviation calculations are feasible since the update rate of the proposed camber inspection system is about 350 measurements/s. Let *N* be the number of data, $C_{i}$ be the *i*-th evaluated camber angle, $\bar{C}$ be the mean of the *N* evaluated camber angles, and δ be the standard deviation of these *N* evaluated camber angles. The following definition is formed:

$$\bar{C}=\frac{1}{N}\sum_{i=1}^{N}C_{i}$$

$$\delta =\sqrt{\frac{1}{N}\sum_{i=1}^{N}{\left(C_{i}-\bar{C}\right)}^{2}}$$

**Figure 10:**
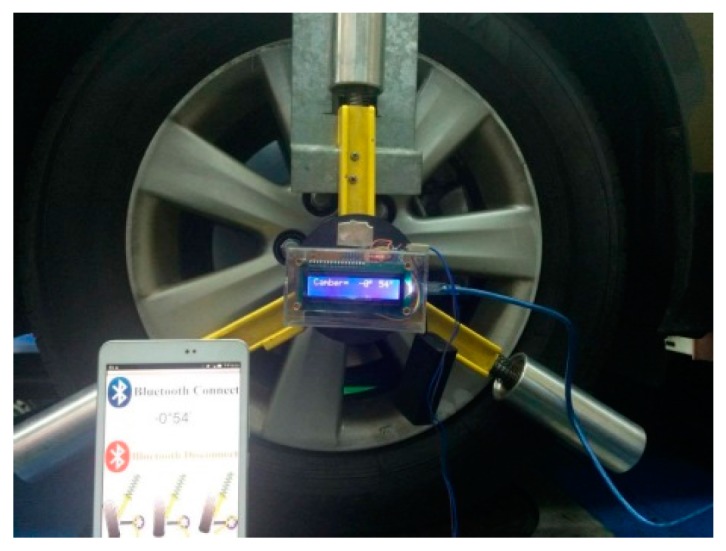
The result evaluated camber angle from the MUC-based system for the front-right wheel of a vehicle.

**Figure 11:**
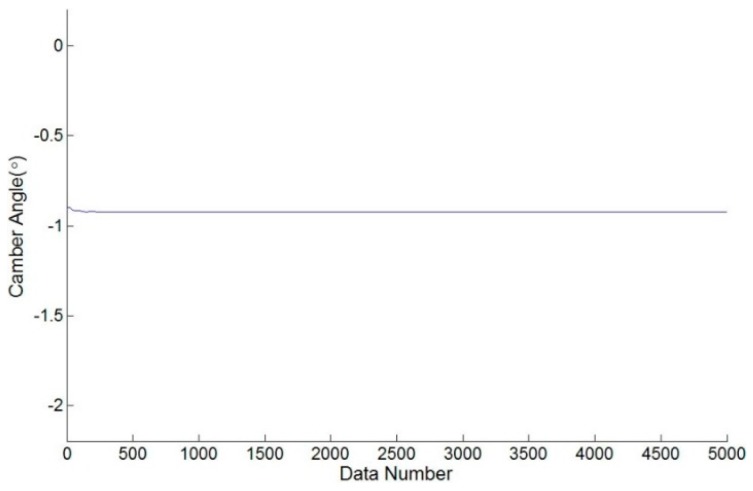
The result camber data inspected from the MCU-based system with a camber angle of $-00^{\circ }54\prime$.

**Figure 12:**
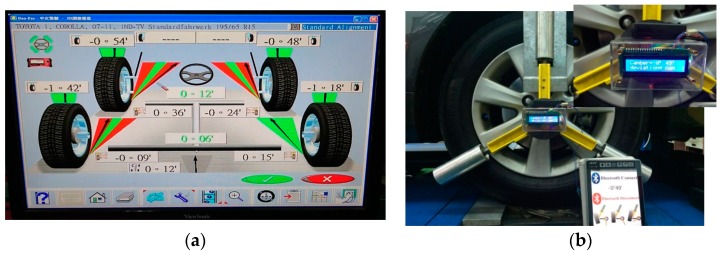
The camber inspection with an iPad display, verified by the vision-based system. (**a**) Results evaluated by the vision-based system; (**b**) results by the MCU-based system.

For a normal distribution, the possibility of the measuring error is about 99.7% within an absolute error of $0.015^{\circ }$ if the deviation of the data is $0.005^{\circ }$, although the distribution of the raw data from the accelerometer may not be a normal distribution. Furthermore, this algorithm is intuitive to implement in MCU. The evaluated camber angle is available if the deviation satisfies the specified value. Otherwise, the evaluated camber angle is not in a settled condition and is unavailable. Figure 10 shows the result of the evaluated camber angle in an authentic camber angle inspection with a specified deviation of $0.005^{\circ }$. The evaluated camber angle data are shown in Figure 11, which shows that the sequential data are smoother than those in Figure 8. In addition, the final evaluated camber angle converges to $-0.9015^{\circ }$ $\left(≅-00^{\circ }54\prime \right)$ within 1200 data, in around 4 s. The different camber measured between the MCU-based system and the vision-based system approximates to $0.0015^{\circ }$. This illustrates that the MCU-based camber inspection system is available for camber angle measurements. Figure 12 shows the results for another authentic camber angle inspection in which the camber angle is $-0.8034^{\circ }$ $\left(≅-00^{\circ }49\prime \right)$ and $-0.8000^{\circ }$ $\left(-00^{\circ }48\prime \right)$ evaluated respectively by the MCU-based system and the vision-based system. The difference measured between the MCU-based system and the vision-based system is usually within $\pm 0.005^{\circ }$. Accordingly, the MCU-based camber inspection system is applicable in the vehicle wheel alignment process.

## 5. Conclusions

This paper has proposed a feasible approach to camber angle inspections for vehicle wheel alignments. The accurate alignment of the *x*-axis for Coordinate *S* and Coordinate *V* is not imperative since the proposed approach compensates for this misaligned angle. This approach will facilitate operations during camber angle measurements of wheels. The proposed approach can directly evaluate the camber angle in Equation (6) with the accelerations in Coordinate *S*. In addition, it can be implemented with an MCU.

Precision is one of the most significant issues if an inspection system can be applied in commercial camber angle measurements. Here, we analyzed the measuring errors for different attitudes between the camber inspection system and the accelerometer. The result shows that it is better to install the axes of the accelerometer aligned instead of those of the camber inspection system. We propose a procedure for calibrating misaligned axes between a camber inspection system and an accelerometer since 3-axis accelerometers cannot normally be installed in exact positions in fabrications of the systems. Calibrations prevent the precision from contaminating the misaligned axes and make the camber inspection system realizable. Furthermore, a signal filter can reduce the sensor noise from camber angle measurements. The total precision is about $\pm 0.005^{\circ }$ for an accelerometer with a sensitivity of 4*mg* in *x-*, *y-*, and *z*-axes. Theoretically, the possibility of precision within $\pm 0.015^{\circ }$ is 99.7% for a normal distribution. The precision is sufficient for the commercial requirement of $\pm 0.02^{\circ }$. The proposed approach is not only feasible but implementable. Two authentic camber angle inspections verify the proposed approach. In addition, the measured data of the camber angle can be transmitted via mediums such as RS232, Bluetooth, and Wi-Fi.

The proposed approach can easily extend to the level gauge since the level gauge has the same mathematical approach, except for the inclinations of different axes. The inspections of another wheel characteristic angle, or toe angle, are also important in wheel alignments. Future studies will focus on the development of the MCU-based toe inspection systems, which will complete measurements for wheel alignments. The reliability of the calibrations for the camber measurement system, which makes the camber measurements more precise and more reliable, is also a significant issue for future studies.

## Abbreviations

The following abbreviations are used in this manuscript:

MCU: micro-control unit
RS232: EIA-RS-232 (Electronic Industry Association Recommended Standard #232)
Wi-Fi: wireless fidelity
